# Protective role of VEGF/VEGFR2 signaling against high fatality associated with hepatic encephalopathy via sustaining mitochondrial bioenergetics functions

**DOI:** 10.1186/s12929-022-00831-0

**Published:** 2022-07-03

**Authors:** Ching-Yi Tsai, Jacqueline C. C. Wu, Chiung-Ju Wu, Samuel H. H. Chan

**Affiliations:** Institute for Translational Research in Biomedicine, Kaohsiung Chang Gung Memorial Hospital, Kaohsiung, Taiwan

**Keywords:** Acute liver failure, Primary neuronal culture, Mitochondrial membrane potential, Bioenergetics failure, Apoptosis, Baroreflex dysregulation

## Abstract

**Background:**

The lack of better understanding of the pathophysiology and cellular mechanisms associated with high mortality seen in hepatic encephalopathy (HE), a neurological complication arising from acute hepatic failure, remains a challenging medical issue. Clinical reports showed that the degree of baroreflex dysregulation is related to the severity of HE. Furthermore, mitochondrial dysfunction in the rostral ventrolateral medulla (RVLM), a key component of the baroreflex loop that maintains blood pressure and sympathetic vasomotor tone, is known to underpin impairment of baroreflex. Realizing that in addition to angiogenic and vasculogenic effects, by acting on its key receptor (VEGFR2), vascular endothelial growth factor (VEGF) elicits neuroprotection via maintenance of mitochondrial function, the guiding hypothesis of the present study is that the VEGF/VEGFR2 signaling plays a protective role against mitochondrial dysfunction in the RVLM to ameliorate baroreflex dysregulation that underpins the high fatality associated with HE.

**Methods:**

Physiological, pharmacological and biochemical investigations were carried out in proof-of-concept experiments using an in vitro model of HE that involved incubation of cultured mouse hippocampal neurons with ammonium chloride. This was followed by corroboratory experiments employing a mouse model of HE, in which adult male C57BL/6 mice and VEGFR2 wild-type and heterozygous mice received an intraperitoneal injection of azoxymethane, a toxin used to induce acute hepatic failure.

**Results:**

We demonstrated that VEGFR2 is present in cultured neurons, and observed that whereas recombinant VEGF protein maintained cell viability, gene-knockdown of *vegfr2* enhanced the reduction of cell viability in our in vitro model of HE. In our in vivo model of HE, we found that VEGFR2 heterozygous mice exhibited shorter survival rate and time when compared to wild-type mice. In C57BL/6 mice, there was a progressive reduction in VEGFR2 mRNA and protein expression, mitochondrial membrane potential and ATP levels, alongside augmentation of apoptotic cell death in the RVLM, accompanied by a decrease in baroreflex-mediated sympathetic vasomotor tone and hypotension. Immunoneutralization of VEGF exacerbated all those biochemical and physiological events.

**Conclusions:**

Our results suggest that, acting via VEGFR2, the endogenous VEGF plays a protective role against high fatality associated with HE by amelioration of the dysregulated baroreflex-mediated sympathetic vasomotor tone through sustaining mitochondrial bioenergetics functions and eliciting antiapoptotic action in the RVLM.

**Supplementary Information:**

The online version contains supplementary material available at 10.1186/s12929-022-00831-0.

## Background

Acute liver failure is a devastating consequence of hepatotoxic liver injury that can lead to the development of neurological complications called hepatic encephalopathy (HE), which manifests a wide spectrum of neuropsychiatric abnormalities and motor disturbance, varying from subtle cognitive deficits to coma [1, 2]. Patients with acute liver failure generally manifested a poor prognosis; the spontaneous survival rate is < 50% [3]. Without liver transplantation, the mortality rate of patients with HE is 50–90% [4, 5]. Mechanisms that have been suggested to underpin HE include increase of ammonia [6], oxidative stress [7], astrocyte swelling [8], brain edema [9, 10], inflammation [9] and mitochondrial dysfunction [11]. However, none of these mechanisms specifically addresses the pathophysiology and cellular mechanisms associated with the high mortality aspect of HE.

By providing a rapid negative feedback mechanism that dampens fluctuations in blood pressure (BP) and heart rate (HR) induced by environmental insults, baroreflex is the most fundamental mechanism in brainstem cardiovascular regulation. Clinical study [12] showed that the degree of baroreflex dysregulation is related to the severity of HE. Previous investigations [13–15] revealed that bioenergetics failure, loss of electron transport ability within the mitochondrial respiratory chain and apoptosis in the rostral ventrolateral medulla (RVLM), a key component of the baroreflex loop [16], mediate dysregulation of baroreflex-mediated sympathetic vasomotor tone that underlies fatality in animal models of brainstem death. It is therefore of interests that we demonstrated previously that defunct baroreflex-mediated sympathetic vasomotor tone is also causally related to fatality in animal models of HE [17].

In addition to its well-known angiogenic and vasculogenic effects, vascular endothelial growth factor (VEGF), which was identified approximately 35 years ago [18, 19], also exhibits neuroprotective effects in the central and peripheral nervous system. Earlier work showed that VEGF protects cells against death from hypoxic injury [20], ischemic stroke [21] and excitotoxic stimuli [22]. Subsequent mechanistic studies demonstrated that VEGF sustains cell survival through regulation of mitochondrial function in human umbilical vein endothelial cells [23, 24]. Depletion of VEGF in endothelial cells results in mitochondrial fragmentation and suppression of glucose metabolism, leading to cell death [24]. Anti-VEGF cancer therapy causes significant reduction of mitochondrial Complex I and II-dependent respiration in cardiomyocytes and loss of mitochondrial membrane potential in endothelial cells [25] or sensory ganglion neurons [26]. VEGF receptor 2 (VEGFR2), also known as kinase insert domain-containing receptor (KDR) or fetal liver kinase-1 (Flk-1) [19], is considered the major mediator of VEGF effects through the activation of a number of signaling pathways [27]. For example, VEGF protects neurons from ischemia [20] via VEGFR2. Our previous work also demonstrated that VEGF sustains myocardial performance via VEGFR2 in the heart [28] and facilitates baroreflex via VEGFR2 in the nucleus tractus solitarii [29].

Arising from the foregoing narrative is the notion, which forms the guiding hypothesis of the present study, that the VEGF/VEGFR2 signaling plays a protective role against mitochondrial dysfunction in the RVLM, which leads to baroreflex dysregulation that underpins the high fatality associated with HE. Based on results from proof-of-concept experiments using an in vitro model of HE, and corroboratory experiments employing an animal model of HE, this hypothesis is validated.

## Methods

### Primary neuronal culture and in vitro model of hepatic encephalopathy

Primary neuronal cultures were prepared from C57BL/6 mice as described previously [30] with modifications. Briefly, the hippocampi were dissected from postnatal day 1 mouse pups and incubated in 1 mg/mL papain solution (#P4762, Sigma-Aldrich, St. Louis, MO, USA) at 37 °C for 10 min. 5 μg/mL DNase I (#DN25, Sigma-Aldrich) was then added to the dissociation mixture for an additional incubation of 5 min. The cell suspension was washed, pelleted, and seeded at 1–2 × 10^6^ cells/well onto 6-well plates for mRNA experiments, 4 × 10^4^ cells/well onto 96-well plates for cell viability assay, or 3–4 × 10^5^ cells/coverslip onto glass coverslips in 12-well plates for immunofluorescence staining. All wells or coverslips were coated in advance with 0.05% poly ethyleneimine (#P3143, Sigma-Aldrich). The cells were cultured in Neurobasal-A medium (#10888022, Gibco, Waltham, MA USA), supplemented with 2% B27 supplement (#17504044, Gibco), 0.5 mM GlutaMAX (#35050061, Gibco), 1% penicillin/streptomycin (#15070063, Gibco) and 4.5 mM HEPES (#15630080, Gibco) and were incubated at 37 °C in a humidified 5% CO_2_ incubator; media were changed every 2–3 days. After 7 days, cultured neurons that reached 70% confluent were used for further experiments. To generate the in vitro model of HE, cultured mouse hippocampal neurons were incubated with ammonium chloride (NH_4_Cl; #A9434, Sigma-Aldrich) for 24 h at a final concentration of 5 mM. This concentration has been used as the experimental input in reported in vitro models of HE [31, 32], and is similar to 5.4 mM found in brain tissue in an in vivo model of HE [33].

### Immunofluorescence staining in cells

Cultured hippocampal neurons were fixed on coverslips with a Fixation Buffer (#420801, BioLegend, San Diego, CA, USA), permeabilized with an Intracellular Staining Permeabilization Wash Buffer (#421002, BioLegend), then blocked with 0.2% Triton X-100, 3% fetal bovine serum (#26140087, Gibco) and 1% DMSO. Immunofluorescence staining was carried out by incubation for 48 h at 4 °C using a rabbit polyclonal antiserum against VEGFR2 (1:250; #ab2349, Abcam, Cambridge, MA, USA) and a mouse monoclonal antiserum against β-Tubulin III (1:1000; #T8578, Sigma-Aldrich). This was followed by incubation with a secondary goat anti-rabbit IgG conjugated with Alexa Fluor 488 (1:500; #A11034, Invitrogen, Eugene, OR, USA) and a donkey anti-mouse IgG conjugated with Alexa Fluor 568 (1:500; #A10037, Invitrogen) for 1 h at room temperature. Nuclei were stained in a commercial mounting medium that contains 0.0002% DAPI (#ab104139, Abcam). Slides were viewed under an Olympus FV10i confocal microscope (Olympus, Tokyo, Japan). FV10i-SW software, 60× objective and 405, 473 and 559 nm laser lines were used for image acquisition; immunoreactivity for β-Tubulin III exhibited red fluorescence, and VEGFR2 exhibited green fluorescence.

### WST-1 cell viability assay

Cell viability was evaluated using the Cell Proliferation Reagent WST-1 (#11644807001, Roche, Mannheim, Germany), which is based on the cleavage of tetrazolium salt by cellular mitochondrial dehydrogenase to form a dark red formazan dye. The amount of formazan dye generated is directly proportional to the number of living cells. Briefly, after replacing the incubation medium with diluted WST-1 Reagent in fresh growth medium, primary cultured neurons were incubated at 37 °C for 4 h. Absorbance was then measured at 450 nm with the reference wavelength at 650 nm using a Varioskan LUX multimode microplate reader (Thermo Scientific, Waltham, MA, USA).

### Recombinant VEGF treatment

Recombinant mouse VEGF protein (rmVEGF; #493-MV-025) was purchased from R&D systems (Minneapolis, MN, USA). VEGF was reconstituted at 50 μg/mL in sterile PBS containing 0.1% BSA and stored at − 20 °C in 50-μL aliquots to minimize damage due to freezing and thawing. Stock solutions were diluted in treatment medium to attain the experimental concentrations prior to the experiments. Cultured neurons were treated with rmVEGF (1 or 5 ng/mL), alone or together with 5 mM NH_4_Cl, for 24 h.

### siRNA transfection

As in our previous study [34], ON-TARGET *plus* SMART pool for mouse VEGFR2 siRNA (#L-040634-00-0020) and Non-Targeting Pool siRNA (#D-001810-10-05) were obtained from Dharmacon (Cambridge, UK). The siRNA was resuspended in 1× siRNA buffer, which was diluted from 5× siRNA buffer (Dharmacon) with RNase-free water, to obtain a 20 μM stock concentration. Cultured neurons were transfected with Lipofectamine RNAiMAX reagent (#13778-075, Invitrogen) according to the manufacturer’s transfection protocol; a final volume 0.3 μL/well in 96-well plate or 7.5 μL/well in 6-well plate was used. Briefly, the cultured neurons were incubated with the siRNA-duplex-Lipofectamine RNAiMAX complex at a final siRNA concentration of 10 nM for 48 h at 37 °C in a CO_2_ incubator.

### Experimental animals and mouse model of hepatic encephalopathy

Adult male C57BL/6 mice were purchased from the National Laboratory Animal Center of the Ministry of Science and Technology, Taiwan. VEGFR2 deficient mice (B6.129-Kdr^tm1Jrt^/J, strain number: 002938) with a targeted mutation (Kdr targeted mutation 1) on a C57BL/6 genetic background purchased from Jackson Laboratory (Bar Harbor, ME, USA) were maintained as heterozygous (KDR^+/−^) and wild-type (KDR^+/+^) colonies; KDR null mice have an embryonic lethal phenotype. Animals were housed in an AAALAC International-accredited facility with maintained room temperature (24 ± 1 °C) and 12 h light/dark cycle (light on at 05:00), and were provided with freely accessible mouse chow and water. All experiments were carried out in accordance to the guidelines for animal experimentation endorsed by the Institutional Animal Care and Use Committee of the Kaohsiung Chang Gung Memorial Hospital (approval number: 2016113003). As recommended by the International Society for Hepatic Encephalopathy and Nitrogen Metabolism Commission guidelines on animal models of HE [35], a single intraperitoneal (i.p.) injection of azoxymethane (AOM; 100 μg/g in sterile saline; #A5486, Sigma-Aldrich), an active metabolite of the cycad palm nut that is hepatotoxic and could be used as a fulminant hepatic failure-inducing hepatotoxin [36], was delivered to induce acute liver failure in mice. Animals without any treatment served as the sham-controls.

### Radiotelemetric recording of cardiovascular parameters

Similar to our previous study [17], BP of mice was recorded continuously over 24 h under a conscious state using implantable blood pressure telemeters (TA11PA-C10; Data Sciences International, St. Paul, MN, USA). The transmitted BP signals were digitized and processed by an arterial blood pressure analyzer (APR31a, Notocord-hem; Instem, Staffordshire, UK) based on feature extraction. Systolic blood pressure (SBP), mean arterial pressure (MAP) and HR was derived from the BP waveforms on a beat-by-beat basis. Continuous, on-line, and real-time spectral analysis (SPA10a, Notocord-hem) of SBP signals was used to detect temporal fluctuation in the low-frequency (BLF; 0.15–0.6 Hz) band, an index for baroreflex-mediated sympathetic vasomotor tone [37]. Concurrent 24-h changes in MAP, HR, power density of the BLF band and activity of the animals were continuously recorded. The averaged values of those parameters recorded every hour were taken to represent the hourly results.

### Intracerebroventricular (i.c.v.) infusion of anti-VEGF antiserum by osmotic minipump

I.c.v. infusion of anti-VEGF antiserum (VEGF Ab; #ab1316, Abcam) or mouse IgG (#sc-2025, Santa Cruz Biotechnology, Dallas, TX, USA) was diluted in artificial cerebrospinal fluid (aCSF) and delivered by an osmotic minipump (Alzet 1007D; Durect Corp, Cupertino, CA, USA) for at least 3 days at a rate of 0.5 μL/h; the amount of antibody was infused at 0.004 μg/h. Control infusion of aCSF (NaCl 117 mM, KCl 4.7 mM, NaH_2_PO_4_ 1.2 mM, MgCl_2_ 1.2 mM, CaCl_2_ 2.5 mM, NaHCO_3_ 25 mM, and glucose 11 mM) served as the volume and vehicle control. 0.02% Triton X-100 was added to facilitate transport of VEGF antiserum or mouse IgG across the cell membrane of neurons [38].

### Collection of tissue samples

Following the procedure in our previous study [17], which is based on temporal changes of baroreflex-mediated sympathetic vasomotor tone, tissue samples from the RVLM were routinely collected at 2, 12 or 24 h after the injection of AOM. Animals were perfused with warm saline, and the brain was rapidly removed and immediately frozen on ice. Tissues from both sides of the RVLM were collected and stored immediately in liquid nitrogen. RVLM tissues collected from anesthetized animals but without treatment with AOM served as the sham-controls.

### Isolation of RNA and real-time PCR

Total RNA from RVLM tissue was extracted by a commercial kit (#RT300, Geneaid, Taipei, Taiwan) and quantified by the ratio of absorbance at 260 nm and 280 nm using a NanoDrop spectrophotometer (Thermo Scientific). Reverse transcriptase reaction was performed using a PrimeScript RT Reagent kit (#RR037A, Takara, Shiga, Japan). The *vegfr2* or *gapdh* gene expression was quantified using TaqMan probe and StepOnePlus Real-time PCR system (Thermo Scientific).

### Protein extraction and Western blot analysis

Total protein from the RVLM was extracted with a tissue protein extraction buffer, which contains a proprietary detergent in 25 mM bicine, 150 mM sodium chloride (pH 7.6; #78510, Thermo Scientific) and centrifuged at 10,000×*g* at 4 °C for 10 min. The concentration of protein was determined by the Pierce BCA protein assay (#23225, Thermo Scientific). Western blot analysis was carried out on VEGFR2 (1:1000; #9698, Cell Signaling Technology, Danvers, MA, USA) and β-actin (1:5000; #MAB1501, EMD Millipore, Temecula, CA, USA). We detected specific antibody-antigen complex using enhanced chemiluminescence reagents in conjunction with UVP BioSpectrum Imaging Systems (Analytik Jena, Upland, CA, USA). Protein expression was presented as a ratio relative to β-actin protein.

### Measurement of ATP content and apoptotic cell death

Tissue samples from the RVLM were similarly homogenized in tissue protein extraction buffer (#78510, Thermo Scientific) and were subjected to measurement of total ATP levels using an ATP detection assay kit (#700410, Cayman, Ann Arbor, MI, USA) according to the manufacturer’s instructions. Light emitted from a luciferase-mediated reaction and measured by a luminometer (Berthold Centro LB 960, Bad Wildbad, Germany) was used to calculate the measured values. Apoptotic cell death in homogenized RVLM tissues was determined following the instructed protocol of a cell death detection kit (#11544675001, Roche). This assay is based on the quantitative sandwich-enzyme immunoassay-principle using mouse monoclonal antibodies directed against DNA and histones to measure the level of cytoplasmic histone-associated DNA fragments as an index of the induced apoptotic cell death. Absorbance was measured at 405 nm using a Varioskan LUX multimode microplate reader (Thermo Scientific).

### Flow cytometry

Fresh tissue samples from the RVLM were washed with 1X PBS and incubated with 0.25% trypsin–EDTA for 30 min at 37 °C with gentle agitation. Samples were gently mixed with tapping by fingers, pipetting and upside-down mixing every 10 min to obtain a cell suspension. Digestion was terminated by MEM medium that contains 5% fetal bovine serum and filtered through cell strainers with 70 μm pores. This was followed by centrifugation at 420×*g* for 5 min and washed twice with 1× PBS. Cells were stained with 5 μM JC-1 (#T3168, Invitrogen), an indicator of mitochondrial membrane potential, for 30 min at room temperature. JC-1 dye exhibits potential-dependent accumulation in mitochondria, indicated by a fluorescence emission shift from green (monomer) to red (aggregate). These changes were detected by a Gallios flow cytometer and analyzed with the Kaluza software (Beckman Coulter, Indianapolis, IN, USA), with analysis of each sample set to 10,000 events.

### Double immunofluorescence staining

Double immunofluorescence staining of frozen transverse section of the medulla oblongata was performed as reported previously [39]. In brief, free-floating 25-μm sections of the medulla oblongata were incubated with a rabbit polyclonal antiserum directed against VEGFR2 (1:250; #ab2349, Abcam), together with a mouse monoclonal antiserum directed against a specific neuron marker, neuron-specific nuclear protein (NeuN; 1:1000; #MAB377, Millipore). The sections were subsequently incubated concurrently with two appropriate secondary antisera, a goat anti-rabbit IgG conjugated with Alex Fluor 488 (1:500; #A11034, Invitrogen) for VEGFR2 and a goat anti-mouse IgG conjugated with Alexa Fluor 568 (1:500; #A11031, Invitrogen) for NeuN. Viewed under Olympus FV1000 confocal microscope (Olympus) using FV10-ASW 4.2 software, 40× objective and 488 and 559 nm laser lines for image acquisition, immunoreactivity for NeuN exhibited red fluorescence, and VEGFR2 exhibited green fluorescence.

### Statistical analysis

All values are expressed as mean ± SEM. One-way analysis of variance was used to assess group means, followed by the Dunnett or Tukey’s post hoc multiple-range test for assessment of individual means. Student’s t-test was used for data from two experimental groups. p < 0.05 was considered statistically significant.

## Results

### Recombinant VEGF protein maintains cell viability in in vitro model of HE

**Fig. 1:**
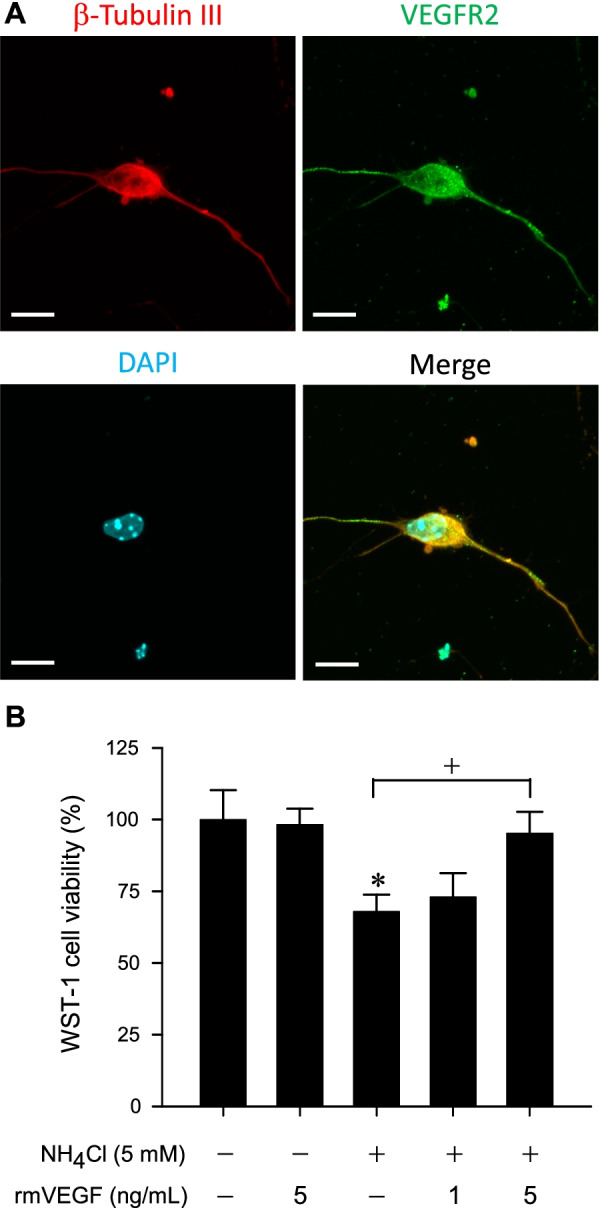
**A** Representative immunofluorescence images showing cells from primary hippocampal neuron culture that were immunoreactive to the neuronal marker β-Tubulin III (red fluorescence) and were additionally stained positively for VEGFR2 (green fluorescence). Nuclei were stained with DAPI (blue fluorescence). Scale bar, 10 µm. **B** Effects of treatment with recombinant mouse VEGF protein (rmVEGF; 1 or 5 ng/mL) on cell viability measured by the WST-1 assay in cultured mouse neurons incubated with or without ammonia (NH_4_Cl; 5 mM). Values are mean ± SEM of 3–4 independent experiments. *p < 0.05 versus control (non-treatment) group in the Dunnett multiple-range test; ^+^p < 0.05 versus NH_4_Cl treatment group in the Tukey’s multiple-range test

The first part of our study was designed to prove the concept that VEGF/VEGFR2 signaling plays a protective role against mitochondrial dysfunction that underpin fatality associated with HE using an in vitro model. As a fundamental prerequisite, our immunofluorescence staining results (Fig. 1A) first established that VEGFR2 is present in the primary cultured hippocampal neurons. On treatment with 5 mM NH_4_Cl [31, 32] to mimic hyperammonemia, a major pathogenesis in the development of HE [6], results from WST-1 assay (Fig. 1B) showed that these neurons exhibited a reduction of cell viability. The indicated decline in mitochondrial dehydrogenase activity implies that mitochondrial bioenergetics failure has taken place. Whereas 1 ng/mL was ineffective, co-incubation with recombinant VEGF protein (5 ng/mL) significantly reversed the reduction of cell viability under 5 mM NH_4_Cl (Fig. 1B). Treatment with recombinant VEGF protein (5 ng/mL) alone did not significantly affect cell viability when compared to non-treatment group (Fig. 1B).

### Gene knockdown of VEGFR2 enhances the reduction of cell viability in in vitro model of HE

**Fig. 2:**
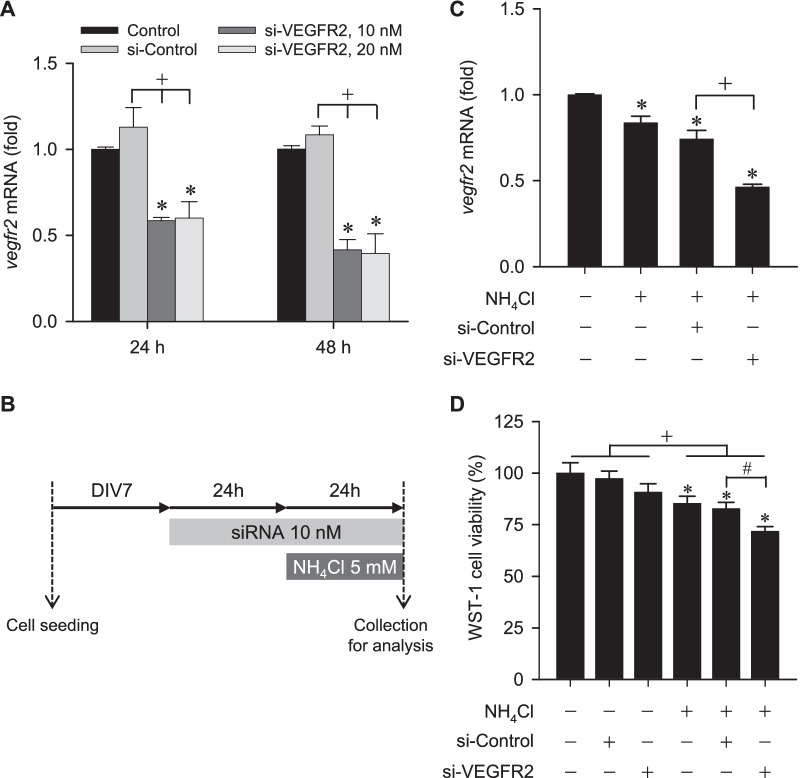
**A** Fold-changes relative to control group of *vegfr2* mRNA expression in cultured mouse neurons detected at different time-points (24 or 48 h) after treatment with different concentrations (10 or 20 nM) of *vegfr2* specific siRNA (si-VEGFR2) or control nonspecific siRNA (si-Control; 20 nM). Values are mean ± SEM of 3 independent experiments. *p < 0.05 versus control (non-treatment) group in the Dunnett multiple-range test; ^+^p < 0.05 versus si-Control group in the Tukey’s multiple-range test. **B** Schematic diagram of siRNA treatment experiments. DIV, days in vitro. **C** Effects of treatment with si-VEGFR2 or si-Control on fold-changes relative to control (non-NH_4_Cl treatment) group of *vegfr2* mRNA expression in cultured neurons incubated with ammonia (5 mM NH_4_Cl). Values are mean ± SEM of 3–4 independent experiments. *p < 0.05 versus non-treatment group in the Dunnett multiple-range test; ^+^p < 0.05 versus si-Control group in the Tukey’s multiple-range test. **D** Effects of treatment with si-VEGFR2 or si-Control on cell proliferation measured by the WST-1 assay in cultured neurons incubated with or without ammonia. Values are mean ± SEM of 3–4 independent experiments. *p < 0.05 versus non-treatment group in the Dunnett multiple-range test; ^+^p < 0.05 versus non-NH_4_Cl treatment group at corresponding group in the Tukey’s multiple-range test; ^#^p < 0.05 versus si-Control treatment group in the Tukey’s multiple-range test

Our next series of experiments employed siRNA for gene knockdown to further ascertain the suggested protective role of VEGF/VEGFR2 signaling in cell viability of the hippocampal neurons under NH_4_Cl treatment. To establish the effective concentration and treatment time (Fig. 2A), we found that transfection with VEGFR2 specific siRNA (si-VEGFR2) induced maximal decrease in *vegfr2* mRNA in cultured neurons 48 h after application when compared to treatment with non-specific siRNA (si-Control). Since both concentrations of VEGFR2 siRNA (10 and 20 nM) induced similar knockdown effects, we have chosen to incubate the hippocampal neurons for 48 h with 10 nM siRNA, in the presence or absence of 5 mM NH_4_Cl for 24 h (Fig. 2B). Compared to si-Control, treatment with si-VEGFR2 exacerbated the significant decrease in *vegfr2* mRNA (Fig. 2C) and reduction in cell viability (Fig. 2D) induced by NH_4_Cl. On the other hand, treatment with si-Control or si-VEGFR2 alone (Fig. 2D) did not significantly affect cell viability when compared to non-treatment group.

### VEGFR2 is present in RVLM neurons and is reduced in mouse model of HE

**Fig. 3:**
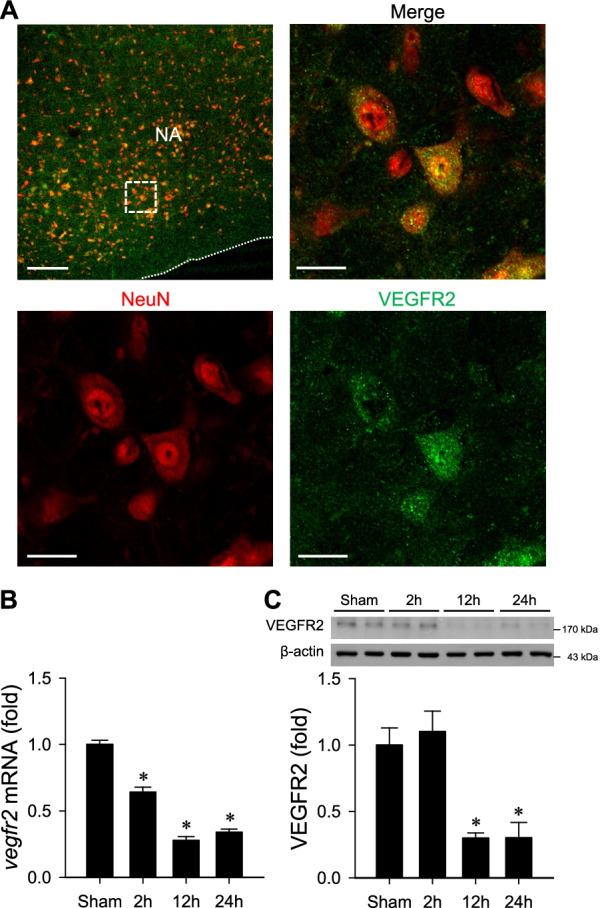
**A** Representative laser scanning confocal microscopic images from the RVLM showing cells that were immunoreactive to a neuronal marker, neuron-specific nuclear protein (NeuN; red fluorescence), and were additionally stained positively for VEGFR2 (green fluorescence). White dotted box in low-power view of the medulla oblongata indicated the location for high-power magnification. Scale bars, 200 μm (low-power view); 20 μm (high-power view). NA, nucleus ambiguous. **B**, **C** Fold-changes relative to sham-controls of *vegfr2* mRNA (**B**) or VEGF protein (**C**) level in the RVLM in mice 2, 12 or 24 h after administration of azoxymethane (AOM; 100 μg/g, i.p.). Values are mean ± SEM, n = 3 animals per group. *p < 0.05 versus corresponding sham-controls group in the Dunnett multiple-range test

The second part of our study was designed to extend the affirmative results from our proof-of-concept in vitro experiments to validate that VEGF/VEGFR2 signaling indeed plays a protective role against mitochondrial dysfunction in the RVLM that underpin high fatality associated with HE, using the AOM-treatment mouse model. Again, we first determined the presence of VEGFR2 in the RVLM, an essential premise for VEGF to play a protective role by modulation of baroreflex. Results from immunofluorescence staining (Fig. 3A) revealed that VEGFR2 immunoreactivity is present in RVLM cells that stained positively with the neuronal marker, NeuN. Real-time PCR (Fig. 3B) and western blot (Fig. 3C) analyses further showed a significant decrease of *vegfr2* mRNA and VEGFR2 protein levels in the RVLM 2–24 h after administration of AOM (100 μg/g, i.p.).

### VEGF/VEGFR2 signaling in the RVLM plays a protective role during experimental HE

**Fig. 4:**
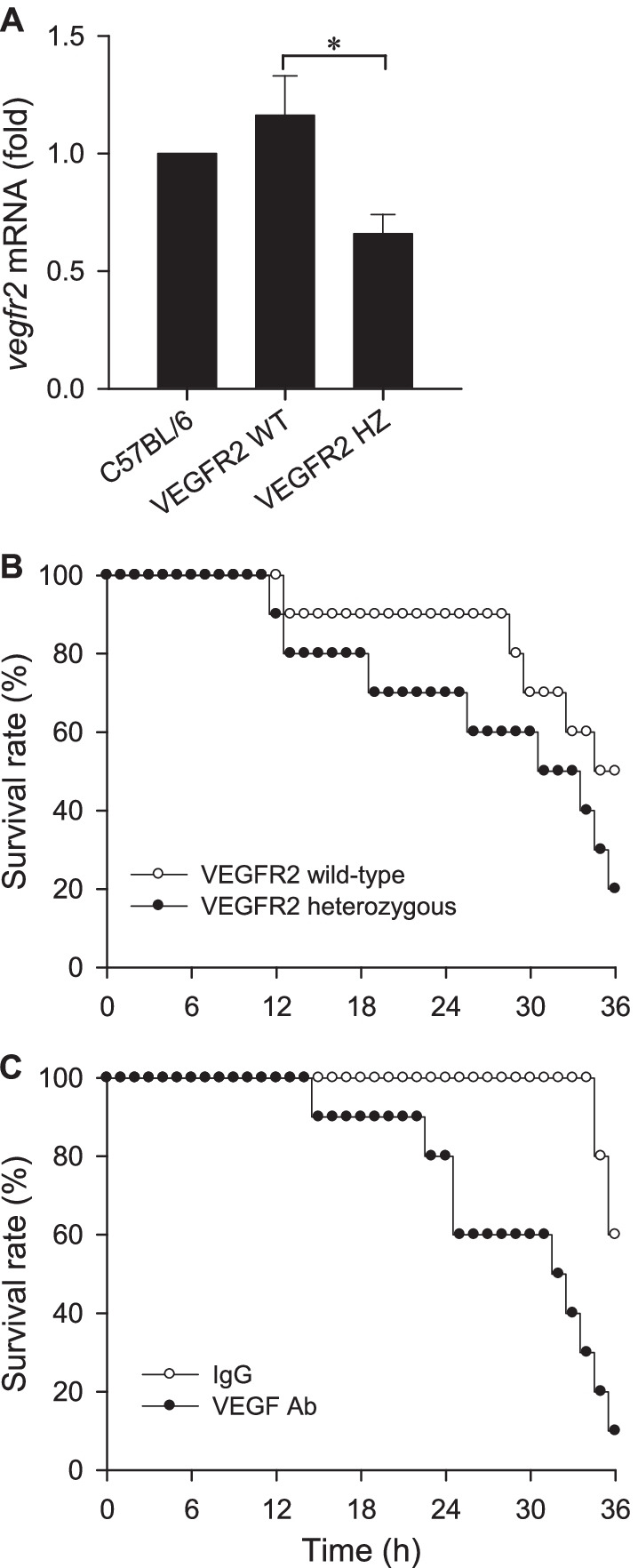
**A** Fold-changes relative to C57BL/6 mice of *vegfr2* mRNA in the RVLM in VEGFR2 wild-type (WT) or heterozygous (HZ) mice. Values are mean ± SEM, n = 4 animals per group. *p < 0.05 versus wild-type mice in the Student’s t-test. **B**, **C** Survival rate over 36 h following i.p. administration of AOM in VEGFR2 wild-type and heterozygous mice (**B**), or in C57BL/6 mice that received pretreatment by i.c.v. infusion of an anti-VEGF antiserum (0.004 μg/h) or mouse IgG (0.004 μg/h) (**C**). n = 10 animals per experimental group

We next established that VEGF/VEGFR2 signaling plays a protective role against fatality in our animal model of HE. On top of a significant reduction in *vegfr2* mRNA level in the RVLM when compared to wild-type mice (Fig. 4A), VEGFR2 heterozygous mice exhibited lower survival rate over 36 h after i.p. injection of AOM (100 μg/g) (Fig. 4B). These observations were confirmed by loss-of-function manipulation of endogenous VEGF. I.c.v. infusion of an anti-VEGF antiserum in C57BL/6 mice (Fig. 4C) also exacerbated the reduction of survival rate induced by AOM in mice treated with mouse IgG. The mean survival time in the anti-VEGF antiserum group was significantly shorter than that in the IgG group (Table 1).

**Table 1:** Comparison of mean survival time in different mouse groups after AOM (100 μg/g, i.p.) injection

Group	Mean survival time (h)	p value
VEGFR2 wild-type	35.5 ± 3.5 (n = 10)	
VEGFR2 heterozygous	28.5 ± 3.6 (n = 10)	p = 0.1805
C57BL/6 (i.c.v.)		
IgG	38.5 ± 1.7 (n = 10)	
VEGF Ab	29.1 ± 2.3 (n = 10)*	p = 0.0041

Values are mean ± SEM

*p < 0.05 versus corresponding control group in the Student’s t-test

### VEGF protects baroreflex dysregulation during experimental HE

**Fig. 5:**
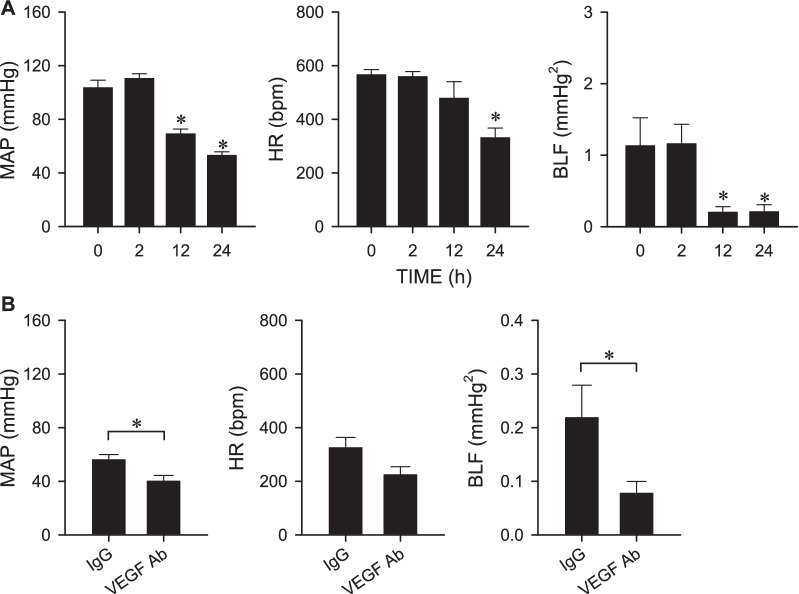
**A** Temporal changes in mean arterial pressure (MAP), heart rate (HR) or power density of the low-frequency (BLF) component of systolic blood pressure spectrum measured by radiotelemetry in mice before (0 h) and 2, 12 or 24 h after administration of AOM (100 μg/g, i.p.). Values are mean ± SEM, n = 6 animals per experimental group. *p < 0.05 vs. 0 h group in the post hoc Dunnett multiple-range test. **B** Changes in MAP, HR or BLF power 24 h after administration of AOM (100 μg/g, i.p.) in mice that received pretreatment by i.c.v. infusion of mouse IgG (0.004 μg/h) or VEGF Ab (0.004 μg/h). Values are mean ± SEM, n = 7 animals per experimental group. *p < 0.05 versus IgG group in the Student’s t-test

As reported previously [17], defunct baroreflex-mediated sympathetic vasomotor tone, leading to hypotension, is causally related to fatality in the AOM mouse model of HE. It is therefore of interest to investigate whether baroreflex dysfunction is the target of the protective actions of VEGF/VEGFR2 signaling. Results from radiotelemetric recording in C57BL/6 mice showed significant reductions in MAP, HR and an index of baroreflex-mediated sympathetic vasomotor tone (BLF power) 24 h after AOM injection (Fig. 5A). Compared to the IgG control group, immunoneutralization of VEGF significantly potentiated the elicited hypotension and depressed baroreflex-mediated sympathetic vasomotor tone at this time-point (Fig. 5B) when 40% of mice succumbed to AOM injection (Fig. 4C).

### VEGF sustains ATP production and diminishes apoptotic cell death in the RVLM during experimental HE

**Fig. 6:**
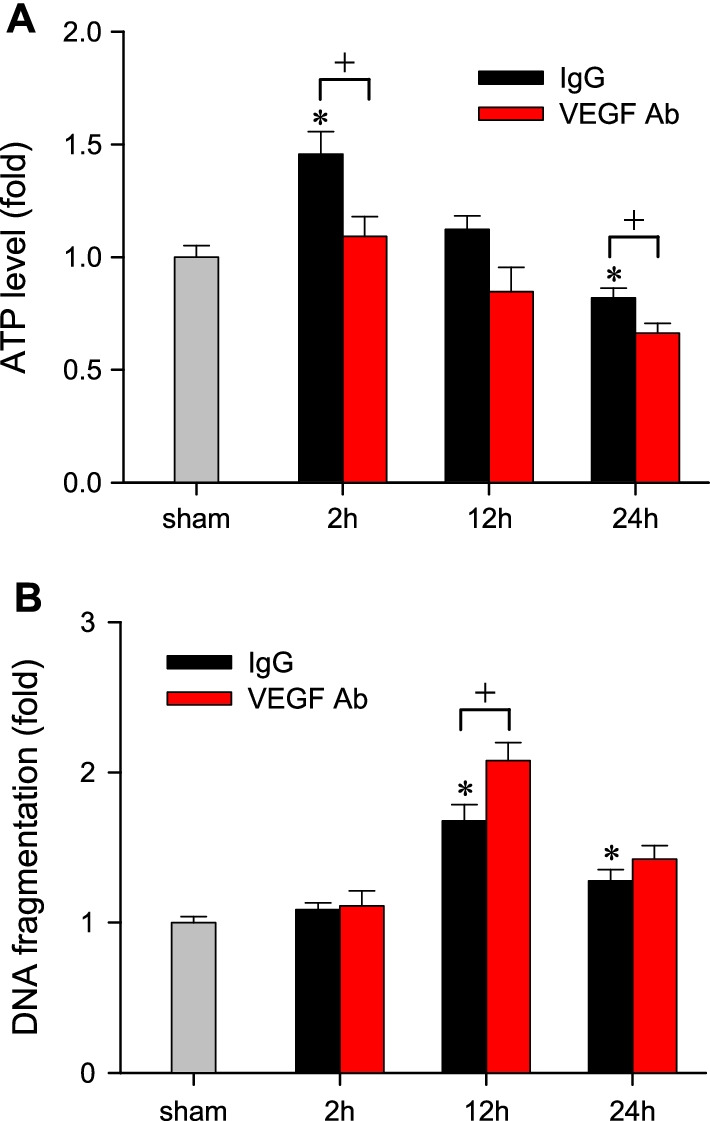
Fold-changes relative to sham-controls in ATP level (**A**) or apoptotic cell death determined by cytoplasmic histone-associated DNA fragments (**B**) in tissues collected from the RVLM in mice that received pretreatment by i.c.v. infusion of mouse IgG (0.004 μg/h) or VEGF Ab (0.004 μg/h) 2, 12 or 24 h after administration of AOM (100 μg/g, i.p.). Samples from sham-controls were obtained randomly at corresponding time-points. Values are mean ± SEM, n = 4 animals per group. *p < 0.05 versus sham-controls group in the Dunnett multiple-range test; ^+^p < 0.05 versus IgG group at corresponding time-points in the Tukey’s multiple-range test

In an attempt to search for the mechanistic basis of the protective actions of VEGF, we found that ATP production in the RVLM underwent a significant increase at 2 h, followed by a decrease at 24 h after AOM injection (Fig. 6A). At the same time, the level of histone-associated DNA fragments, a marker of apoptosis, in the RVLM exhibited a significant augmentation at 12 h and 24 h (Fig. 6B). Immunoneutralization of VEGF significantly retarded the changes in ATP production (Fig. 6A) and exacerbated apoptotic cell death manifested 12 h (Fig. 6B) after treatment with AOM.

### Endogenous VEGF maintains mitochondrial membrane potential in the RVLM in mouse model of HE

**Fig. 7:**
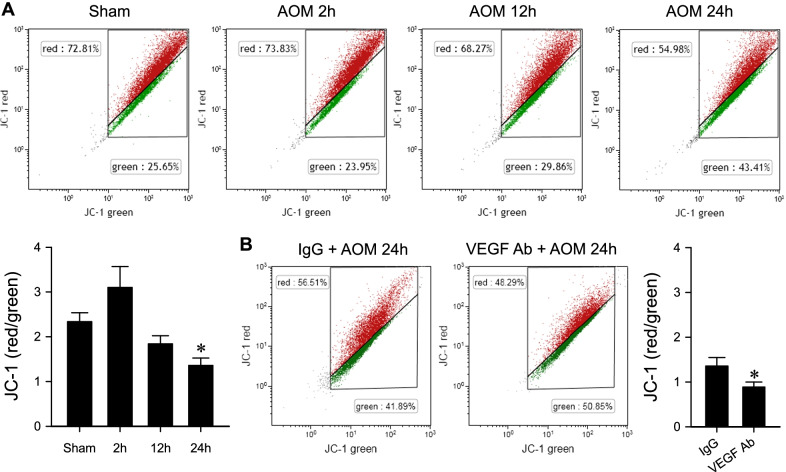
**A** Changes in the ratio of aggregated (red fluorescence indicating high mitochondrial membrane potential) to monomer (green fluorescence indicating low mitochondrial membrane potential) JC-1 in cells isolated from the RVLM under 2, 12 or 24 h after administration of AOM (100 μg/g, i.p.). Samples from sham-controls were obtained randomly at corresponding time-points. Values are mean ± SEM, n = 5 animals per experimental group. *p < 0.05 versus sham-controls group in the Dunnett multiple-range test. **B** Effects of pretreatment by i.c.v. infusion of mouse IgG (0.004 μg/h) or VEGF Ab (0.004 μg/h) on the decrease of mitochondrial membrane potential detected by JC-1 24 h after AOM injection in C57BL/6 mice. Values are mean ± SEM, n = 5 animals per group. *p < 0.05 versus IgG groups in the Student’s t-test

As a mitochondrial membrane potential indicator, JC-1 is predominantly a monomer that yields green fluorescence at low mitochondrial membrane potential and becomes aggregated to yield a red to orange fluorescence at high mitochondrial membrane potential. Figure 7A showed a significant decrease in the aggregate fluorescent count in the RVLM, indicating a decrease of mitochondrial membrane potential during HE. We also found that i.c.v. infusion of an anti-VEGF antiserum significantly enhanced the decrease of mitochondrial membrane potential exhibited 24 h after AOM injection in C57BL/6 mice when compared to the IgG control group (Fig. 7B).

## Discussion

Because of the high mortality, HE remains a challenging clinical issue in contemporary medicine. In particular, the lack of better understanding of the pathophysiology and cellular mechanisms associated with this aspect of HE further aggregates the problem. Based on physiological, pharmacological and biochemical observations from proof-of-concept experiments using an in vitro model of HE and corroboratory experiments employing an animal model of HE, the present study fills this void of knowledge. Our results suggest that the VEGF/VEGFR2 signaling plays a protective role against mitochondrial dysfunction in the RVLM, leading to amelioration of baroreflex dysregulation that underpins high fatality associated with HE.

Our data from immunofluorescence, PCR, western blot and gain-of-function or loss-of-function experiments demonstrated that VEGF, acting via VEGFR2, plays a protective role in both our cell and animal models of HE using primary hippocampal neurons and C57BL/6 and heterozygous or wild-type VEGFR2 mice. In particular, results from WST-1 assay in cultured neurons and JC-1 assay in dispersed RVLM cells showed that this protective action is exerted via mitigation of the elicited mitochondrial dysfunction during HE.

Hyperammonemia has been strongly implicated in the pathogenesis of HE [6, 40]. Impairment of the detoxification processes during liver failure results in increased blood levels of ammonia, which readily crosses the blood–brain barrier (BBB) and accumulates in the central nervous system where it evokes neuropsychiatric abnormalities during HE [41]. Two most often mentioned mechanisms for the elicited ammonia toxicity are dissipation of the mitochondrial membrane potential indicative of mitochondrial bioenergetics failure [42, 43] and induction of mitochondrial permeability transition that leads to apoptosis [42, 44]. Involvement of the first mechanism is demonstrated by the declined mitochondrial dehydrogenase activity that is indicative of failure in mitochondrial electron transport chain in our in vitro model of HE employing incubation of cultured hippocampal neurons with NH_4_Cl. Preliminary results showed an increase in ammonia level in the RVLM (1.97 versus 2.41 nmol/mg in sham-control and 12 h after AOM administration). Under this condition, both mechanisms are exhibited by the reduced mitochondrial membrane potential, which indicates mitochondrial bioenergetics failure and changes in ATP level, alongside the occurrence of apoptosis and necrosis (Additional file 1: Fig. S1) in the RVLM in our animal model of HE. We are aware that additional mechanisms, including astrocyte swelling [8], brain edema [9, 10], oxidative stress [7], inflammation [9] and mitochondrial dysfunction [11], may also contribute to HE. Furthermore, AOM has been reported to disrupt the BBB independent from triggering acute liver failure [45]. Breakdown of the BBB should therefore be included as another contributing factor to HE.

The ammonia level in brain in animal models of HE is still a matter of debate. Cooper [46] reported that the normal concentration of ammonia in rat brain is 0.18 mM, rising to 0.42 mM in urease-treated rats or 0.82 mM in l-methionine-*S*,*R*-sulfoximine-treated rats in those two acute hyperammonemia models. On the other hand, 5.4 mM ammonia was found in brain tissue in another in vivo model of HE [33]. This concentration is comparable with our in vitro model of HE in which cultured neurons were incubated with ammonium chloride at a final concentration of 5 mM.

A decrease in VEGFR2 expression that accompanied the decrease in mitochondrial membrane potential in cultured neurons or induction of apoptosis in RVLM tissue samples, along with exacerbation of these events by loss-of-function manipulation of VEGFR2, strongly suggest a protective role for VEGF/VEGFR2 signaling in experimental HE. Such a protective action may be executed via enhancement of mitochondrial bioenergetics [23, 47] or prevention of induction of mitochondrial permeability transition [48]. Results from our mouse model further revealed that the VEGF/VEGFR2 signaling exerts its protective action by acting on the RVLM via both modes of execution to ameliorate the dysregulated baroreflex-mediated sympathetic vasomotor tone, leading to hypotension that is causally related to fatality associated with HE.

As the major source of reactive oxygen species (ROS) in the brain [49], one consequence of deficiency in mitochondrial electron transport chain is generation of ROS, particularly superoxide anion [50]. It thus is of interest that augmented superoxide production in the RVLM has been demonstrated to underpin the cardiovascular dysregulation in our mouse of HE [17]. It follows that the VEGF/VEGFR2 signaling may exert its protective effects by sustaining the integrity of brainstem cardiovascular regulation through reduction of ROS production via facilitation of mitochondrial electron transport chain activities [25].

The brain is highly dependent on energy for normal activity, and mitochondria are the major source of ATP production. As such, the parallel time-course increases of ATP production and mitochondrial membrane potential in the RVLM at 2 h after AOM administration, followed by decreases at 24 h are of relevance. In particular, antagonism of these parallel events by immunoneutralization of VEGF is intriguing. These observations suggest that the VEGF/VEGFR2 signaling sustains baroreflex-mediated sympathetic vasomotor tone and BP via augmented mitochondrial bioenergetics in the RVLM during the initial stage of experimental HE. However, in the face of significant bioenergetics failure and the drastically reduced VEGFR2 in the RVLM, this protective action of VEGF is significantly retarded.

Our results showing that treatment with VEGF antiserum exacerbated apoptotic cell death in the RVLM suggest that VEGF may exert its protection on brainstem cardiovascular regulation via an antiapoptotic action. Neurons also tend to undergo necrosis in response to ATP deficiency [51]. Indeed, coincidental to significant decrease in ATP level in the RVLM, hematoxylin and eosin staining (Additional file 1: Fig. S1) showed necrosis-appearing neurons in this brainstem site that were characterized by eosinophilicity (shrunken cell body, darkly stained red eosinophilic cytoplasm, and lacking discernible nucleolus) 24 h after AOM injection. That immunoneutralization of VEGF further reduced ATP production at this time-point therefore suggests that the VEGF/VEGFR2 signaling also exerts its protective action via amelioration of necrotic cell death in the RVLM.

VEGF has been reported to act as an autocrine neuroprotective factor in retinal ganglion cells [52]. In the present study, we found that knockdown of VEGFR2 in cultured neurons slightly reduced, albeit without statistical significance, cell viability in the absence of ammonia (90.7 ± 4.16%, p = 0.276). Furthermore, knockdown of VEGFR2 significantly exacerbated ammonia-induced reduction of cell viability (Fig. 2D). These observations together suggest that interaction of autocrine expression of endogenous VEGF with VEGFR2 leads to neuroprotection. However, a recent study using a rat model of minimal HE [53] reported that the autocrine VEGF induces inflammation and oxidative stress through VEGFR2 and COX-2 interaction in astrocytes and causes impairment of neuronal survival. Whether VEGF/VEGFR2 signaling has opposing roles in different cell types (astrocyte versus neuron) or different HE models (minimal HE versus HE) should be clarified in the future.

We noted that whereas treatment with an anti-VEGF antiserum in C57BL/6 mice effected a significant decrease in survival time (p = 0.0041) after AOM administration, reduction of the same in VEGFR2 heterozygous mice was not statistically significant (P = 0.1805) when compared to the wild-type mice. One possible reason is that instead of VEGFR2 null mice, which are embryonic lethal, VEGFR2 deficient mice maintained as a heterozygous colony were used. These mice exhibited a reduction of only 43% of *vegfr2* mRNA in the RVLM against the wild-type mice. We also observed that the magnitude of survival rate 36 h post-AOM injection in IgG-treated C57BL/6 mice was different from VEGFR2 wild-type mice (60% versus 50%) although both mouse strains share the same genetic background. Given that 10 mice were initially used in each group, this disparity may have been amplified because the actual difference is only one animal.

We also recognize three potential limitations in the present study. First, the biological actions of VEGF are mediated by at least two tyrosine kinase receptors: VEGFR1 and VEGFR2 [54]. Since our study design did not include examining the potential involvement of VEGFR1 in the protective actions of VEGF on RVLM neurons, future explorations are needed to address this issue. Second, treatments with an anti-VEGF antiserum were delivered by osmotic minipump via the i.c.v. route. That the RVLM is an action target is demonstrated by the significant treatment effects of immunoneutralization of VEGF on changes in ATP levels, DNA fragmentation and JC-1 manifested in RVLM tissues (Figs. 6 and 7). Site-specific microinjection of anti-VEGF antiserum into the RVLM is required to exclude the possibility of secondary effects. Third, in a chronic study that lasted 32 weeks [55], AOM was found to induce histopathological changes in liver and kidney of Swiss albino mice. The possibility therefore exists for our observed fatality to be related to direct AOM-induced hepatic and renal toxicity. Demonstration of biochemical and histological changes in liver and kidney in our acute model of HE will resolve this possibility.

## Conclusions

Based on physiological, pharmacological and biochemical observations from proof-of-concept experiments using an in vitro model of HE and corroboratory experiments employing an animal model of HE, the present study demonstrated that VEGF plays a protective role against high fatality associated with HE. Mechanistically, we showed that acting via VEGFR2, the endogenous VEGF ameliorates the dysregulated baroreflex-mediated sympathetic vasomotor tone, which underlies the elicited fatality, by sustaining mitochondrial bioenergetics functions and eliciting antiapoptotic action in the RVLM.

## Supplementary Information

**Additional file 1: Figure S1.** Histopathology of the RVLM during HE. Low-power (**A**–**C**) or high-power (**D**–**F**) photomicrographs showing typical histological changes in the RVLM stained with hematoxylin and eosin 12 or 24 h after mice received AOM administration. White dotted box in low-power view of the medulla oblongata indicated the location for high-power magnification. Note in **F** irreversibly damaged neurons (yellow arrows). The cell body was shrunken and displayed intensively eosinophilic cytoplasm; the nucleus was pycotic and lacked discernible nucleolus. Scale bar, 100 μm in low-power or 50 μm in high-power photomicrographs. *NA* nucleus ambiguus.

## Data Availability

The datasets generated during and/or analyzed during the current study are available from the corresponding author on reasonable request.

## Abbreviations

aCSF: Artificial cerebrospinal fluid
AOM: Azoxymethane
BBB: Blood–brain barrier
BLF: Low-frequency component of systolic blood pressure spectrum
BP: Blood pressure
Flk-1: Fetal liver kinase-1
HE: Hepatic encephalopathy
HR: Heart rate
HZ: Heterozygous
I.c.v.: Intracerebroventricular
I.p.: Intraperitoneal
KDR: Kinase insert domain-containing receptor
MAP: Mean arterial pressure
NH_4_Cl: Ammonium chloride
ROS: Reactive oxygen species
RVLM: Rostral ventrolateral medulla
SBP: Systolic blood pressure
si-Control: Non-specific siRNA
si-VEGFR2: *vegfr2* specific siRNA
VEGF: Vascular endothelial growth factor
VEGF Ab: Anti-VEGF antiserum
VEGFR2: VEGF receptor 2
rmVEGF: Recombinant mouse VEGF protein
WT: Wild-type
