# Supplementary material for: Protective role of VEGF/VEGFR2 signaling against high fatality associated with hepatic encephalopathy via sustaining mitochondrial bioenergetics functions
Source: J Biomed Sci. 2022 Jul 3;29:47. doi: 10.1186/s12929-022-00831-0 (PMC9251935; doi:10.1186/s12929-022-00831-0)
Supplement: Supplementary file 1 — Additional file 1: Figure S1. Histopathology of the RVLM during HE. Low-power (A–C) or high-power (D–F) photomicrographs showing typical histological changes in the RVLM stained with hematoxylin and eosin 12 or 24 h after mice received AOM administration. White dotted box in low-power view of the medulla oblongata indicated the location for high-power magnification. Note in F irreversibly damaged neurons (yellow arrows). The cell body was shrunken and displayed intensively eosinophilic cytoplasm; the nucleus was pycotic and lacked discernible nucleolus. Scale bar, 100 μm in low-power or 50 μm in high-power photomicrographs. NA nucleus ambiguus. [file 12929_2022_831_MOESM1_ESM.pdf]

Additional file 1

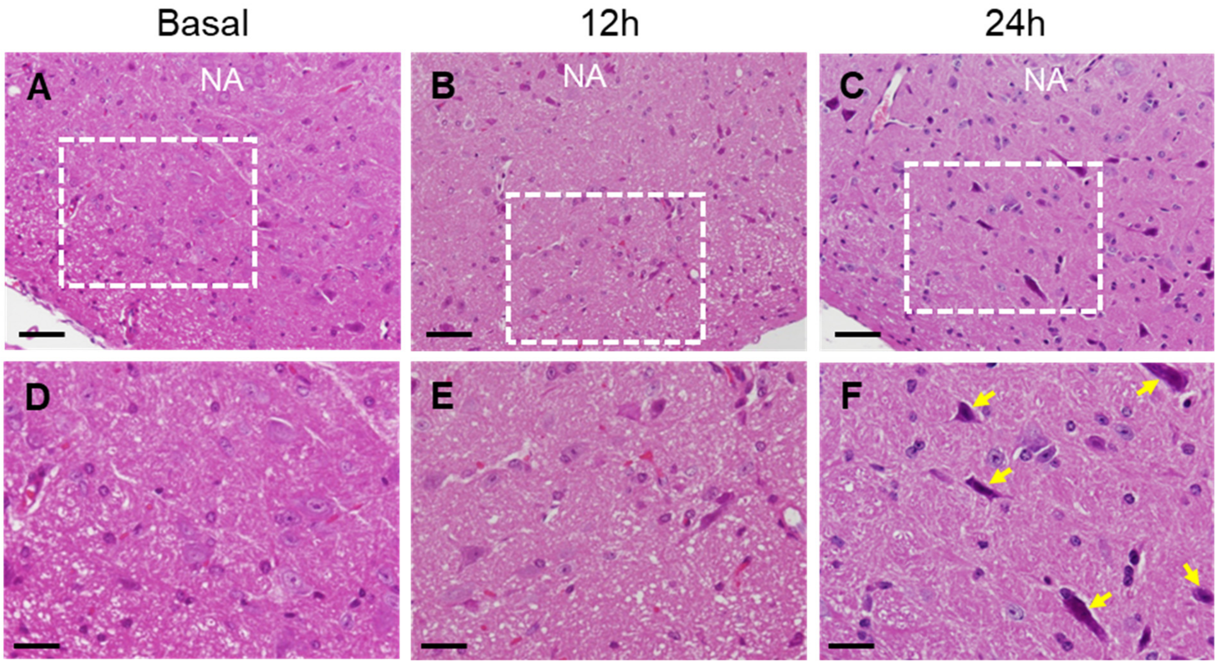

Figure S1. Histopathology of the RVLM during HE. Low-power (**A**-**C**) or high-power (**D**-**F**) photomicrographs showing typical histological changes in the RVLM stained with hematoxylin and eosin 12 or 24 h after mice received AOM administration. White dotted box in low-power view of the medulla oblongata indicated the location of high-power view. Note in (**F**) irreversibly damaged neurons (yellow arrows). The cell body was shrunken and displayed intensively eosinophilic cytoplasm; the nucleus was pycotic and lacked discernible nucleolus. Scale bar, 100 µm in low-power view or 50 µm in high-power view. NA, nucleus ambiguus.
